# Construction of a portable multiplex detection system for four bee viral paralysis diseases based on RT-PCR-microfluidic chip integrated technology

**DOI:** 10.1186/s12917-025-04969-5

**Published:** 2025-08-26

**Authors:** Ziyan Wang, Boyang Xia, Tiantian Fei, Yuming Liu, Zhifeng Gao, Xiuwei Shu, Mingxiao Ma, Dongliang Fei

**Affiliations:** 1 Collaborative Innovation Center for Zoonosis Prevention and Treatment of Jinzhou Medical University, Jinzhou Medical University, Jinzhou, 121001 China; 2 Key Laboratory of Livestock Products Quality and Safety Engineering, Jinzhou Medical University, Jinzhou, 121001 China; 3 College of Animal Husbandry and Veterinary Medicine, Jinzhou Medical University, Jinzhou, 121001 China; 4 Henan University of Chinese Medicine, Zhengzhou, 450046 China; 5 Liaoning center for animal disease control and prevention, Shenyang, 110015 China; 6 Liaoning Yikang Biological CoLtd, Liaoyang, 111000 China

**Keywords:** Bee paralysis disease, Microfluidic technology, Lateral flow dipstick, Polymerase chain reaction, Radid detection

## Abstract

**Background:**

As crucial pollinators sustaining agricultural ecosystem services and biodiversity, bees mediate pollination for approximately 35% of global insect-pollinated crops and generate multidimensional ecological value through apicultural products in the pharmaceutical and food industries. However, emerging viral pathogens pose escalating threats to bee health.

**Results:**

To address the technical bottlenecks in pathogen detection for viral paralysis disease in bees, this study innovatively integrated multiplex RT-PCR amplification, lateral flow dipstick (LFD), and centrifugal microfluidic chip technology (MFCT) to develop an on-site quadruple detection platform capable of simultaneously identifying four viruses: Chronic Bee Paralysis Virus (CBPV), Black Queen Cell Virus (BQCV), Deformed Wing Virus (DWV), and Israeli Acute Paralysis Virus (IAPV). Through multiple sequence alignment, conserved genomic regions of the four viruses were identified, and systematic screening was performed to optimize primer combinations, with critical parameters such as primer concentration (10 µM) and annealing temperature (55 °C) determined. Building on this, a RT-PCR-LFD-MFCT integrated detection system was established by incorporating chemically modified downstream primers/probes and MFCT. Experimental results demonstrated a sensitivity of 10² copies/µL for single-virus detection, enabling precise identification of low viral loads. The method exhibited exceptional specificity with no cross-reactivity, and clinical sample validation achieved 100% concordance with conventional RT-qRT-PCR.

**Conclusions:**

This system features simultaneous multi-target detection, high specificity, rapid processing, minimal instrumentation requirements, portability, and field applicability. It provides a robust tool for precise diagnosis and control of bee paralysis diseases, particularly suitable for resource-limited apiaries and outbreak scenarios, demonstrating significant practical value for safeguarding apicultural health.

**Supplementary Information:**

The online version contains supplementary material available at 10.1186/s12917-025-04969-5.

## Background

As a keystone species sustaining agricultural ecosystems and biodiversity, honeybees serve as critical pollinators for one-third of global pollinator-dependent crops [1, 2], while their products hold immense economic value in pharmaceuticals, food industries, and beyond [3, 4]. However, the worldwide spread of Colony Collapse Disorder (CCD) in recent decades has severely threatened the sustainability of apiculture [5]. Among pathogens contributing to CCD, four viruses stand out as major threats: Chronic Bee Paralysis Virus (CBPV), Deformed Wing Virus (DWV), Israeli Acute Paralysis Virus (IAPV), and Black Queen Cell Virus (BQCV) [6–8]. CBPV induces tremors and paralysis in adult bees, leading to colony collapse [9]; DWV causes wing deformities and developmental defects in juveniles [10]; BQCV destroys queen larvae, disrupting colony succession [11]; and IAPV is characterized by high lethality and acute colony failure [12]. These single-stranded RNA viruses often exhibit synergistic pathogenicity, transmitted via parasitic mites or direct contact, exacerbating colony losses [13, 14]. Notably, their RNA-dependent RNA polymerase (RdRp) genes harbor highly conserved regions across viral genomes, providing both diagnostic targets and insights into viral latency and evolutionary risks [15, 16].

Existing diagnostic approaches encompass biological assays, immunological techniques such as immunocolloidal gold test strips and enzyme-linked immunosorbent assay, and molecular methods including quantitative RT-PCR, conventional RT-PCR, isothermal nucleic acid amplification, and gene sequencing [17–19]. However, these methods are constrained by their reliance on specialized equipment, high operational costs, labor-intensive workflows, and stringent technical expertise requirements. Moreover, their inability to support rapid on-site detection in field settings severely limits their practicality for bee viral paralysis disease monitoring. Consequently, there is an urgent need to develop portable, sensitive, and specific diagnostic solutions tailored for real-world apicultural applications [20, 21].

Microfluidic technology, an interdisciplinary frontier, has revolutionized diagnostics through micron-scale fluidic control, enabling the integration of multi-step biochemical processes onto compact chip platforms [22, 23]. Its advantages—miniaturization, automation, and cost-efficiency—have been demonstrated in medical diagnostics, environmental monitoring, and food safety. Leveraging these advances, this study developed a dual-mode detection system combining laboratory-based assays with field-ready visual diagnostics for four bee viruses [24, 25]. The resulting toolkit not only mitigates disease impacts but also enhances the economic sustainability of beekeeping operations by enabling timely interventions, thereby promoting apicultural health management.

## Results

### Integrated RT-PCR-LFD-MFCT detection system

**Fig. 1:**
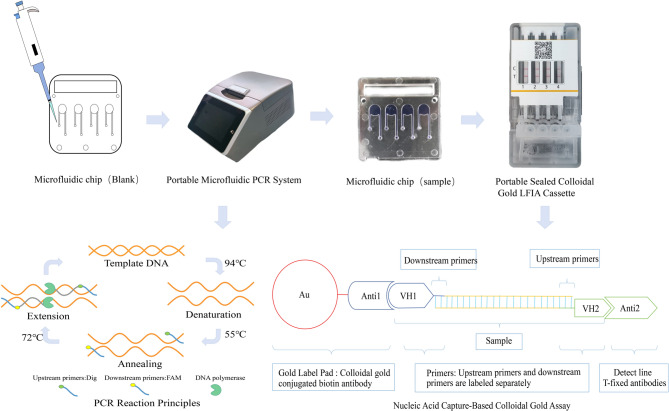
Schematic diagram of the detection system

This study successfully established a visual rapid detection platform by integrating RT-PCR amplification, immunocolloidal gold test strips, and microfluidic chip technologies (Fig. 1). The core of this detection method lies in designing a dual-labeled primer system: the forward primer is modified with digoxigenin (DIG) at its 5’ end, and the reverse primer is modified with carboxyfluorescein (FAM) at its 5’ end. Target nucleic acids are amplified within the micro-reaction chambers of the chip, and the DIG- and FAM-labeled primers are specifically incorporated into the ends of the amplification products. After amplification is complete, the products migrate via capillary action to the detection zone of the test strip. At the conjugate pad, colloidal gold-labeled anti-FAM antibodies are released and bind to the FAM labels on the amplification products, forming a complex. This complex continues to migrate laterally to the test line (T line). On the T line, anti-DIG antibodies are immobilized. When the complex flows through this area, the DIG label at the other end of the amplification product is captured by the anti-DIG antibodies on the T line. This forms a dual-antibody sandwich complex: “immobilized anti-DIG antibody-DIG label-nucleic acid amplification product-FAM label-colloidal gold-labeled anti-FAM antibody”,producing visible red bands for direct interpretation.

### Optimization of the RT-PCR reaction system

#### Optimal primer screening

Agarose gel electrophoresis results under the established RT-PCR system revealed that primer pairs CBPV-F2-R2, BQCV-F2-R2, DWV-F5-R5, and IAPV-F3-R3 produced the clearest target bands without non-specific amplification under identical conditions (Figure S1). These primer pairs were selected as the optimal set for subsequent experiments.

#### Primer concentration optimization

The selected primers were serially diluted to concentrations of 14 µM, 12 µM, 10 µM, 8 µM, 6 µM, and 4 µM for amplification experiments (Figure S2). Comprehensive evaluation across all four viruses revealed that the 10 µM concentration yielded optimal clarity of target bands with the least amount of primer dimer formation. This concentration was thus established as the optimal reaction condition.

#### Optimal annealing temperature

Gradient RT-PCR amplification was performed at annealing temperatures of 60 °C, 58 °C, 55 °C, 53 °C, 50 °C, and 48℃ (Figure S3).Given that the Portable Microfluidic PCR System needs to perform detection reactions for four viruses simultaneously, it is necessary to determine a uniform annealing temperature applicable to all target viruses, It shows that 55 °C produced the most intense bands with negligible non-specific products, confirming this temperature as the optimal annealing condition.

#### Sensitivity assessment

A sensitivity evaluation was conducted on the selected primer sets using viral Plasmid (kindly provided by Dr. Cheng), which was serially diluted across six logarithmic gradients (10^5^–10^0^ copies/µL), with a negative control included. Amplification under optimized RT-PCR conditions revealed a detection limit of 10^1^ copies/µL, as demonstrated (Figure S4), where distinct amplification curves were observed at the 10^1^ copies/µL threshold.

#### Specificity validation

RT-PCR reactions were performed using cDNA templates of four common honeybee viruses: CBPV, BQCV, DWV, and IAPV (Figure S5). Specific amplification curves were observed exclusively for the target viruses, with no cross-reactivity detected for non-target pathogens. This confirms the high specificity of the developed detection method.

### RT-PCR-LFD-MFCT detection primers

The original RT-PCR primers were re-engineered to design RT-PCR-LFD primers and probes. As shown in Figures S1–S4, Primer Set 2 was selected as optimal for CBPV and BQCV, Primer Set 5 for DWV, and Primer Set 3 for IAPV. The four selected primers were modified with digoxigenin (DIG) at the 5′-end of the forward primer and 6-carboxyfluorescein (6-FAM) at the 5′-end of the reverse primer (Table 1). All oligonucleotides were synthesized by Sangon Biotech (Shanghai, China).

**Table 1:** Primer pairs for RT-PCR-LFD-MFCT detection of CBPV, BQCV, DWV, and IAPV

Number	Name	Sequence(5’−3’)
1	CBPV-F2(Dig) CBPV-R2(FAM)	5’-Dig-ACAATGCAAAACCTAGTAACATC−3’ 5’-FAM-CCATCCCATTCTTTGGCAAAAT−3’
2	BQCV-F2(Dig) BQCV-R2(FAM)	5’-Dig-ATGCGCTTTATCGAGGAGGAG−3’ 5’-FAM-TGGAACTCTGCGACTCCCTT−3’
3	DWV-F5(Dig) DWV-R5(FAM)	5’-Dig-TTTGCAAGATGCTGTATGTGG−3’ 5’-FAM-GTCGTGCAGCTCGATAGGAT−3’
4	IAPV-F3(Dig) IAPV-R3(FAM)	5’- Dig-CCATGCCTGGTGATTCAC−3’ 5’- FAM-CTGAATAATACTGTGCGTATC−3’

### RT-PCR-LFD-MFCT sensitivity

**Fig. 2:**
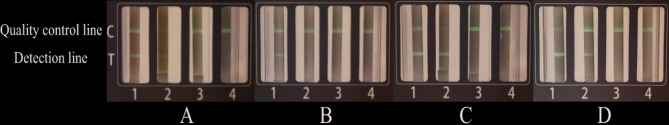
Sensitivity evaluation results of the RT-PCR-LFD-MFCT system. **A **CBPVsensitivity:1:1×10^3^copies/μL;2:1×10^2^copies/μL;3:1×10^1^copies/μL;4:Negativecontrol. **B** BQCVsensitivity:1:1×10^3^copies/μL;2:1×10^2^copies/μL;3:1×10^1^copies/μL;4:Negativecontrol. **C** DWVsensitivity:1:1×10^3^copies/μL;2:1×10^2^copies/μL;3:1×10^1^copies/μL;4:Negativecontrol. **D** IAPVsensitivity:1:1×10^3^copies/μL;2:1×10^2^copies/μL;3:1×10^1^copies/μL;4:Negativecontrol

The standard plasmid was serially diluted in 10-fold increments across three logarithmic gradients and tested under optimized conditions using the detection strips (Fig. 2A-D). No false-positive results were observed when using a 1:1 forward/reverse primer ratio with 0.6 µL primer volume, establishing the method’s limit of detection (LOD) at 10^2^copies/µL.

### RT-PCR-LFD-MFCT specificity

**Fig. 3:**
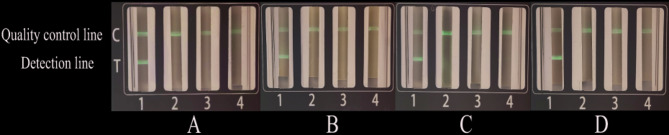
Specificity validation results of the RT-PCR-LFD-MFCT system. **A** CBPV specificity: 1: CBPV; 2: BQCV; 3: DWV; 4: IAPV. **B **BQCV specificity: 1: BQCV;2: CBPV;3: DWV;4: IAPV. **C **DWV specificity: 1: DWV;2: CBPV;3: BQCV;4: IAPV. **D **IAPV specificity: 1: IAPV;2: CBPV;3: BQCV;4: DWV

Under optimal experimental conditions, the specificity of the RT-PCR-LFD-MFCT assay was validated for four honeybee viruses (Fig. 3A-D). Results demonstrated target-specific amplification exclusively for CBPV, BQCV, DWV, and IAPV templates, with no cross-reactivity observed for non-target viruses. These findings align with conventional RT-PCR results, confirming the high specificity of the developed method.

### RT-PCR-LFD-MFCT clinical sample detection results

**Fig. 4:**
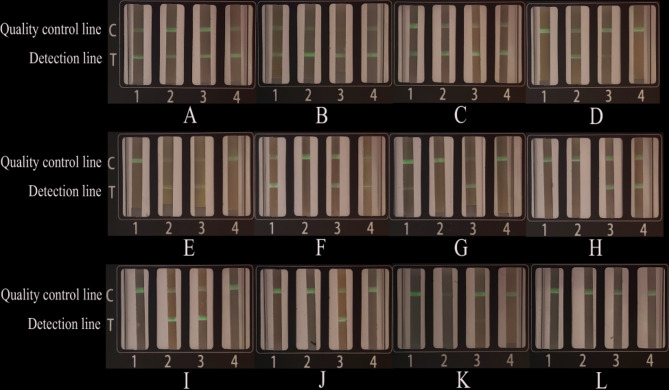
Randomized testing of 12 simulated clinical samples with the RT-PCR-LFD-MFCT system. **A**-**L**. 1: BQCV; 2: CBPV; 3: DWV; 4: IAPV

Twelve suspected infected bee samples from Liaoning were tested using the RT-PCR-LFD-MFCT method, with results from conventional RT-qPCR [26, 27] serving as a comparison (Fig. 4A–L; Table 2). Both methods detected 6 CBPV-positive and 6 negative samples, 5 BQCV-positive and 7 negative samples, 11 DWV-positive and 1 negative sample, and 5 IAPV-positive and 7 negative samples. Mixed infections were identified in 3 cases (all four viruses), 1 case (three viruses), and 5 cases (two viruses), with a 100% concordance rate between the two methods, confirming the reliability of the RT-PCR-LFD-MFCT system for clinical diagnostics.

**Table 2:** Detection in simulated clinical samples using the RT-PCR-LFD-MFCT assay and comparison with RT-qRT-PCR performance

Number	qRT-PCR				RT-PCR-LFD-MFCT			
	BQCV	CBPV	DWV	IAPV	BQCV	CBPV	DWV	IAPV
1	24.628	21.472	11.899	24.628	+	+	+	+
2	22.051	13.844	23.214	14.483	+	+	+	+
3	25.401	8.315	15.706	23.174	+	+	+	+
4	negative	25.177	30.751	negative	-	+	+	-
5	negative	29.437	27.314	Negative	-	+	+	-
6	13.475	negative	21.712	25.111	+	-	+	+
7	21.678	negative	18.899	negative	+	-	+	-
8	negative	negative	19.255	18.432	-	-	+	+
9	negative	28.678	7.903	negative	-	+	+	-
10	negative	negative	8.131	negative	-	-	+	-
11	negative	negative	31.940	negative	-	-	+	-
12	negative	negative	negative	negative	-	-	-	-

Ct mean threshold cycle value of positive samples, ＋ positive detection, － negative detection

## Discussion

As one of the most crucial pollinators in nature, bees play an irreplaceable role in maintaining ecosystem balance and agricultural productivity. In recent years, viral paralysis diseases in bees, characterized by high mortality rates and rapid transmission, have posed severe threats to global honeybee health and emerged as a major pathogen contributing to Colony Collapse Disorder (CCD) [28]. These viruses disrupt the neurological functions of bees, leading to tremors, flight impairment, and abnormal colony behaviors in worker bees, ultimately causing mass mortality [29]. Challenges in detecting bee viral diseases arise from their asymptomatic early stages, prolonged latency, limited availability of specialized diagnostic facilities, and the absence of standardized laboratory methods, resulting in prolonged detection cycles [30]. Therefore, establishing efficient and precise viral detection methods is critical for disease control and ecological conservation.

This study successfully developed a RT-PCR-microfluidic chip integrated method for detecting four bee viral paralysis pathogens (CBPV, BQCV, DWV, and IAPV). RNA extraction and cDNA synthesis were performed using commercial kits. Prepared samples were subsequently loaded into a microfluidic chip, amplified via a Portable Microfluidic PCR System, with results ultimately visualized using a Portable Sealed Colloidal Gold-based LFIA Cassette.The method achieves visible colorimetric results within 5 min, results valid for 10 min, and allows simultaneous identification of all four viruses without specialized equipment.

While the detection method presented in this study generates multiple signals, it differs from traditional multiplex detection techniques. Through specific design within the system, this method achieves individual detection of single viruses. This approach not only enhances detection efficiency but also avoids cross-reactivity, thereby ensuring the accuracy and reliability of the test results.Secondly, RNA was extracted using conventional kits and subsequently synthesized into cDNA. However, rapid, equipment-free RNA extraction methods are currently available. We are exploring the integration of both approaches to enhance the portability of the entire detection system.

Sensitivity analysis demonstrated a detection limit of 10²copies/µL, indicating high sensitivity. Specificity tests confirmed that the method accurately recognized the target viruses without cross-reactivity. However, it should be noted that this validation only covered the four main prevalent strains (CBPV, BQCV, IAPV and DWV) in the western honeybee community and did not include other potentially relevant bee viruses, a limitation that will be addressed in subsequent experiments. In addition, given that there are three main genotypes of DWV, A, B and C, the primers and probes of this method have been specifically optimized for the DWV-A genotype, and their specificity for the DWV-B and DWV-C genotypes has not been systematically evaluated.Clinical validation showed 100% concordance between the newly developed RT-PCR-LFD-MFCT method and conventional RT-qRT-PCR, underscoring its reliability for practical applications.

## Conclusion

In summary, the RT-PCR-based lateral flow dipstick (LFD) and microfluidic chip technology (MFCT) detection platform developed in this study offers high sensitivity, specificity, rapidity, and portability, making it a powerful tool for precise diagnosis and management of bee paralysis diseases. However, further improvements are needed to enhance environmental interference resistance, reduce consumable costs, optimize user-friendliness, and address challenges posed by viral diversity and evolution. Future efforts should focus on refining sample pretreatment protocols, advancing material engineering for detection kits, and fostering interdisciplinary collaboration through bee virus surveillance networks to ensure robust support for sustainable apiculture.

## Methods

### Viruses and clinical samples

The Israeli Acute Paralysis Virus (IAPV), Chronic Bee Paralysis Virus (CBPV), Deformed Wing Virus (DWV), Black Queen Cell Virus (BQCV), and cDNA from healthy bees (free from the four viruses and used as negative controls) were obtained from the specimen repository of the Life Sciences Research Institute at Jinzhou Medical University.The obtained samples were subjected to RNA extraction using the TRANS RNA Extraction Kit (TRANS, Chain), and the extracted RNA was reverse transcribed into cDNA using the Thermo Scientific RevertAid First Strand cDNA Synthesis Kit (Molecular biology, lithuania), and stored at −20℃.

### Primer design

In this study, specific primers (Tables S1-S4) were designed by BLAST comparison based on the sequences of the conserved regions of the RdRp genes of four viruses CBPV(GenBank IDs: KU950353.1), BQCV (GenBank IDs: JX149531), IAPV (GenBank IDs: OR496458.1), and DWV (GenBank IDs: KX373899) obtained from the NCBI database. Among them, the CBPV plasmid (corresponding to the nucleotide segment 1607–1957 of the GenBank IDs: KU950353.1 sequence) was constructed by our laboratory in the previous stage (a gift from Ms. Cheng [31]); while the standard plasmids of BQCV, IAPV and DWV were constructed by this study based on the sequence comparison results of the corresponding strains in the NCBI database (GenBank IDs: MZ821807, GenBank IDs: MG599488.1, GenBank IDs: MZ821820) were constructed from the sequence comparison results of the corresponding strains in the NCBI database (GenBank IDs: JX149531, GenBank IDs: OR496458.1, GenBank IDs: KX373899) (Table S5). All primers and plasmids were synthesized by Sangyo Bioengineering (Shanghai) Co., Ltd. and preserved by dilution with DEPC-treated water after synthesis. The plasmid concentration was determined by Thermo Fisher Scientific (USA) UV-Vis spectrophotometer and calculated by the following. Store the validated plasmids at -20°C for backup.

$$\begin{aligned}\text{equationPlasmid}\ \text{copy}\ \text{number}\ (\text {copies}/ \mu \text L)=&[\text {plasmid}\ \text{concentation}\ (g/\mu\text L)\\&\times10-9\times6.02\times1023]\\&/ \{[\text{Vector}\ \text{length}\ (\text {bp})\\&+\text{fragment}\ \text{length} (\text{bp})]\\&\times 660/\text{gmol}\}\end{aligned}$$

### Optimization of RT-PCR reaction system

The standard RT-PCR reaction mixture was composed as follows: 12.5µL of 2× Basis Taq RT-PCR Mix, 0.5µL each of forward and reverse primers (10µM), 1µL of template DNA, and 10.5µL of DEPC-treated water (total volume: 25µL). Thermal cycling conditions included an initial denaturation at 94 °C for 2 min, followed by 35 cycles of 94 °C for 45 s, 50 °C for 45 s, 72 °C for 45 s, and a final extension at 72 °C for 10 min.Amplification was performed using a thermal cycler (HYBAID, UK). Primers dissolved in RNase-free H₂O were tested using standard plasmid templates. The amplified products were analyzed via 1.2% agarose gel electrophoresis using an automated gel imaging system (Tanon, China) to screen for the optimal primer pair. Subsequent optimizations involved gradient RT-PCR (annealing temperature range: 60–48 °C) and primer concentration gradients (14–4µM) to determine the optimal annealing temperature and working primer concentration. Sensitivity was evaluated using serially diluted templates (10^5^–10^0^copies/µL), while specificity was validated through cross-reactivity tests with non-target pathogens.

### Establishment and optimization of RT-PCR-LFD-MFCT system

A dual-labeled detection system (RT-PCR-LFD) was developed by integrating lateral flow dipstick (LFD) technology with RT-PCR. The selected downstream primer was modified with digoxigenin (DIG) at its 5′-end as a capture marker, while the probe was labeled with FAM fluorophore at its 3′-end as a detection signal. Post-amplification treatment was performed using the protocol described in “The standard RT-PCR reaction mixture” under the section “Optimization of RT-PCR Reaction System”,RT-PCR products were loaded onto a microfluidic chip and inverted onto a fully enclosed colloidal gold test strip cartridge. Results were interpreted after 10 min by observing the test line (T-line) and control line (C-line). The sensitivity of the test strip was assessed using gradient-diluted positive samples (10^3^–10^1^ copies/µL), and specificity was confirmed using nucleic acids from three potential cross-reactive pathogens.

### Clinical validation of RT-PCR-LFD-MFCT

Twelve honeybee samples collected from diverse regions in Liaoning Province were processed for RNA extraction and reverse-transcribed into cDNA. The newly established microfluidic method and a validated RT-qRT-PCR protocol were applied in parallel to evaluate clinical performance. Comparative analysis confirmed the reliability and diagnostic efficacy of the RT-PCR-LFD-MFCT system.

## Supplementary Information

Supplementary Material 1.

Supplementary Material 2.

## Data Availability

The gene sequences used in this study were obtained from NCBI for CBPV (GenBank IDs: KU950353.1), BQCV (GenBank IDs: JX149531), IAPV (GenBank IDs: OR496458.1), and DWV (GenBank IDs: KX373899).
