# Supplementary material for: Construction of a portable multiplex detection system for four bee viral paralysis diseases based on RT-PCR-microfluidic chip integrated technology
Source: BMC Vet Res. 2025 Aug 26;21:524. doi: 10.1186/s12917-025-04969-5 (PMC12379352; doi:10.1186/s12917-025-04969-5)
Supplement: Supplementary file 1 — Supplementary Material 1. [file 12917_2025_4969_MOESM1_ESM.pdf]

The area marked with a dashed red line indicates the cropped portion

Supplementary Figure 1 Primer Screening Results

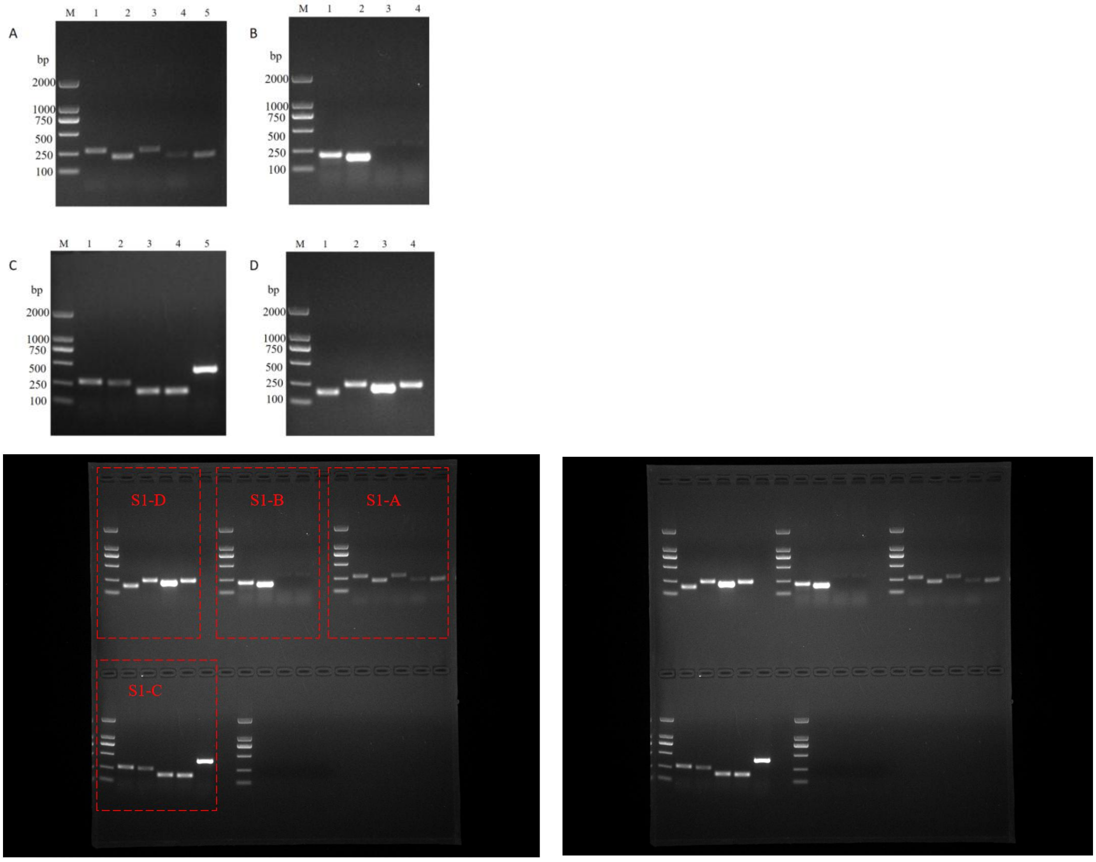

A. CBPV Primer Pair Screening:M: 2000 bp DNA marker; 1: CBPV-F1-R1; 2: CBPV-F2-R2; 3: CBPV-F3-R3; 4: CBPV-F4-R4; 5: CBPV-F5-R5. B. BQCV Primer Pair Screening:M: 2000 bp DNA marker; 1: BQCV-F1-R1; 2: BQCV-F2-R2; 3: BQCV-F3-R3; 4: BQCV-F4-R4. C. DWV Primer Pair Screening:M: 2000 bp DNA marker; 1: DWV-F1-R1; 2: DWV-F2-R2; 3: DWV-F3-R3; 4: DWV-F4-R4; 5: DWV-F5-R5. D. IAPV Primer Pair Screening:M: 2000 bp DNA marker; 1: IAPV-F1-R1; 2: IAPV-F2-R2; 3: IAPV-F3-R3; 4: IAPV-F4-R4.

Supplementary Figure 2 Primer Concentration Optimization Results

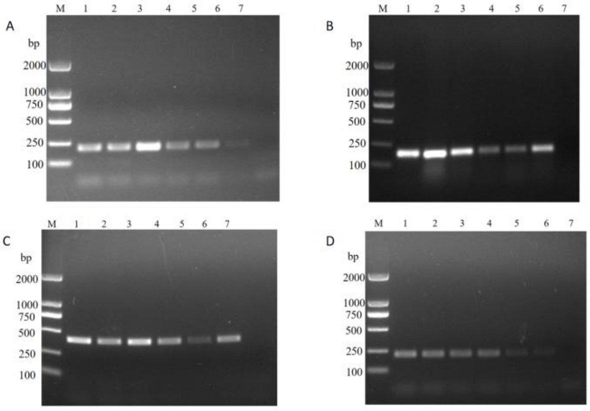

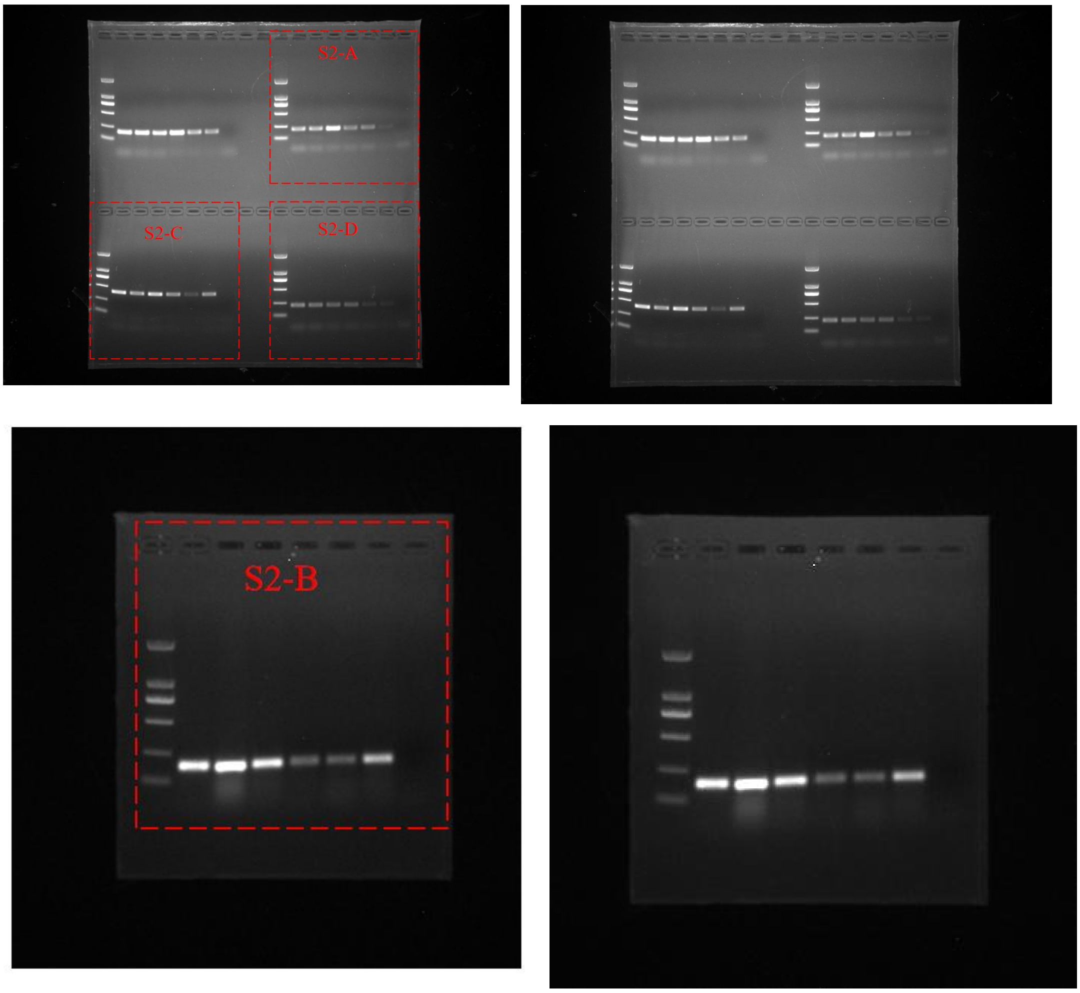

A. CBPV Primer Concentration Screening:M: 2000 bp DNA marker; 1: CBPV-14 μM; 2: CBPV-12 μM; 3: CBPV-10 μM; 4: CBPV-8 μM; 5: CBPV-6 μM; 6: CBPV-4 μM; 7: Negative control. B. BQCV Primer Concentration Screening:M: 2000 bp DNA marker; 1: BQCV-14 μM; 2: BQCV-12 μM; 3: BQCV-10 μM; 4: BQCV-8 μM; 5: BQCV-6 μM; 6: BQCV-4 μM; 7: Negative control. C. DWV Primer Concentration Screening:M: 2000 bp DNA marker; 1: DWV-14 μM; 2: DWV-12 μM; 3: DWV-10 μM; 4: DWV-8 μM; 5: DWV-6 μM; 6: DWV-4 μM; 7: Negative control. D. IAPV Primer Concentration Screening:M: 2000 bp DNA marker; 1: IAPV-14 μM; 2: IAPV-12 μM; 3: IAPV-10 μM; 4: IAPV-8 μM; 5: IAPV-6 μM; 6: IAPV-4 μM; 7: Negative control.

Supplementary Figure 3 Optimal Annealing Temperature Determination

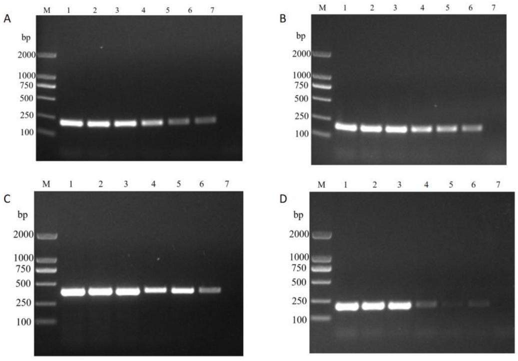

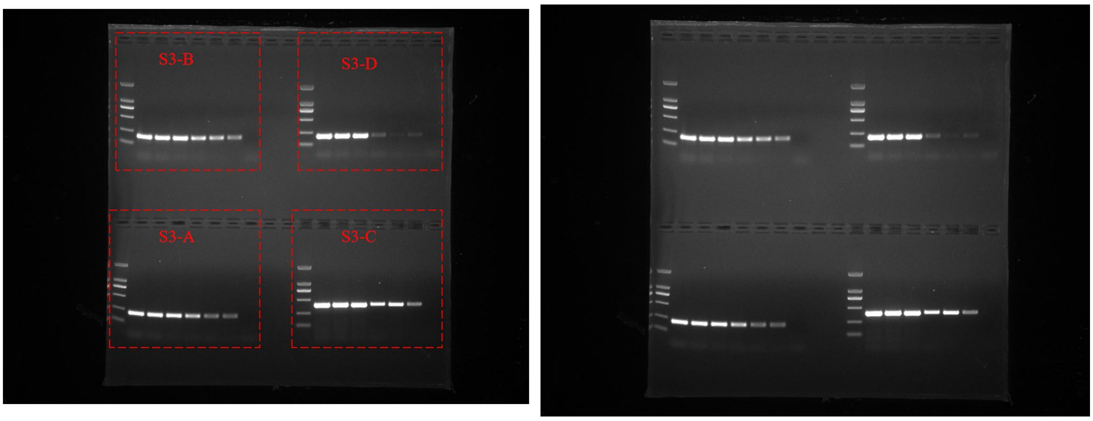

A. Optimal Annealing Temperature for CBPV:M: 2000 bp DNA marker; 1: CBPV-60℃; 2: CBPV-58℃; 3: CBPV-55℃; 4: CBPV-53℃; 5: CBPV-50℃; 6: CBPV-48℃; 7: Negative control. B. Optimal Annealing Temperature for BQCV:M: 2000 bp DNA marker; 1: BQCV-60℃; 2: BQCV-58℃; 3: BQCV-55℃; 4: BQCV-53℃; 5: BQCV-50℃; 6: BQCV-48℃; 7: Negative control. C. Optimal Annealing Temperature for DWV:M: 2000 bp DNA marker; 1: DWV-60℃; 2: DWV-58℃; 3: DWV-55℃; 4: DWV-53℃; 5: DWV-50℃; 6: DWV-48℃; 7: Negative control. D. Optimal Annealing Temperature for IAPV:M: 2000 bp DNA marker; 1: IAPV-60℃; 2: IAPV-58℃; 3: IAPV-55℃; 4: IAPV-53℃; 5: IAPV-50℃; 6: IAPV-48℃; 7: Negative control.

Supplementary Figure 4 Sensitivity Assessment Results

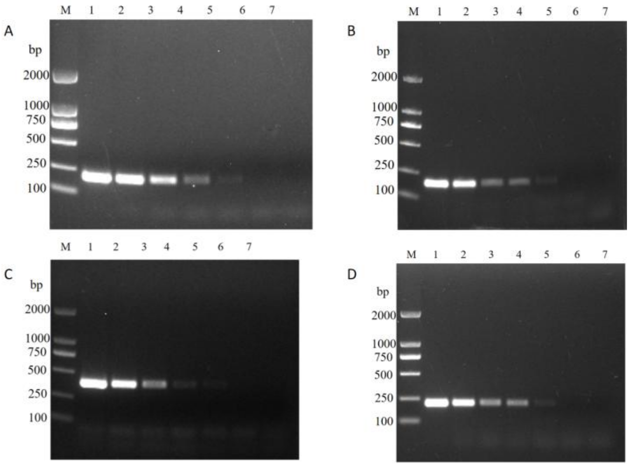

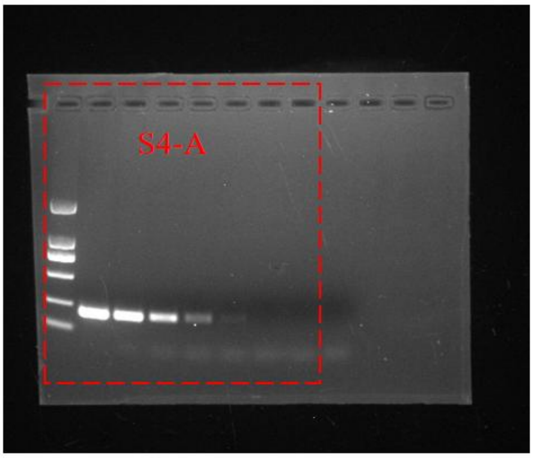
S4-A
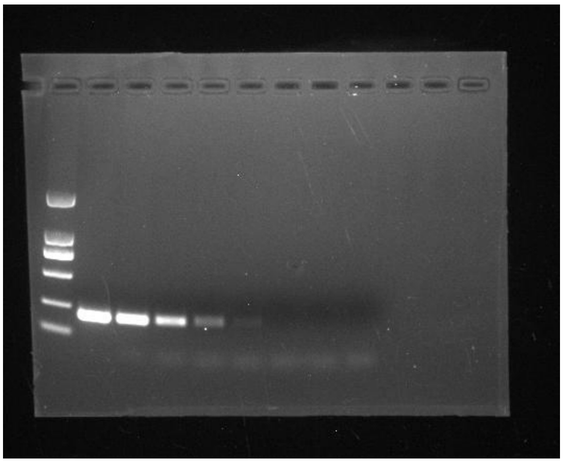
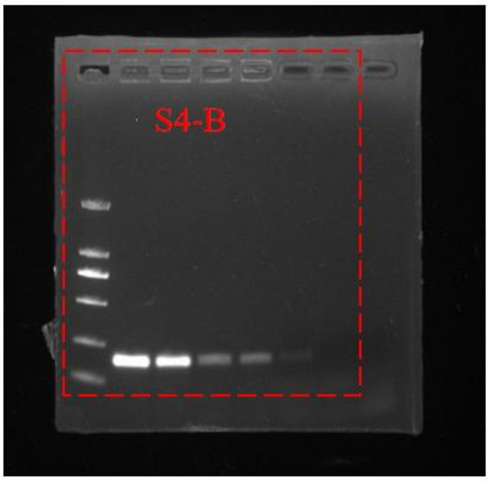
S4-B
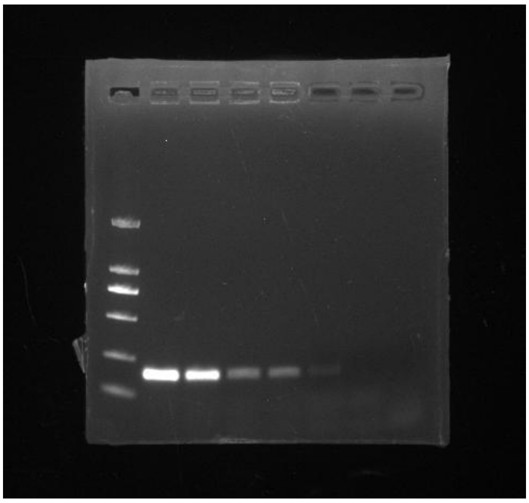
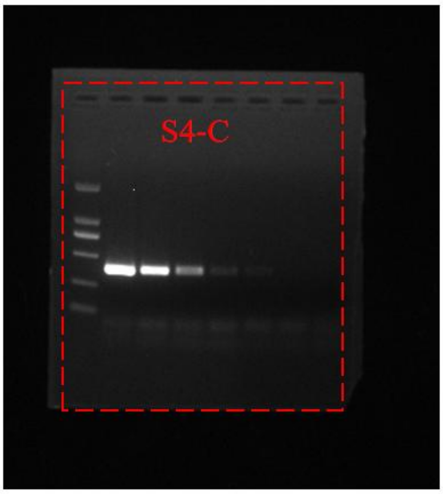
S4-C
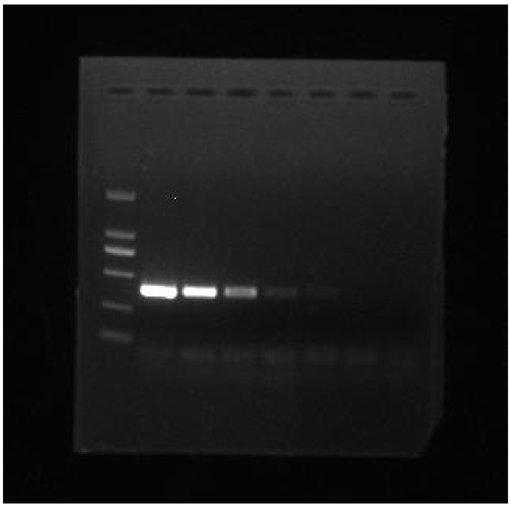

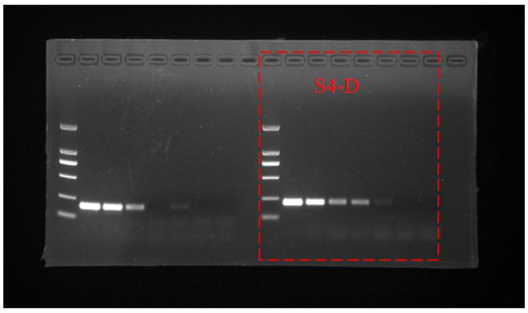

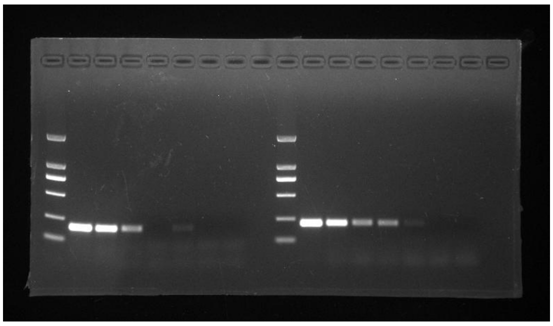

A. CBPV Sensitivity Analysis:M: 2000 bp DNA marker; 1: CBPV-10^5 copies/μL; 2: CBPV-10^4 copies/μL; 3: CBPV-10^3 copies/μL; 4: CBPV-10^2 copies/μL; 5: CBPV-10^1 copies/μL; 6: CBPV-10^0 copies/μL; 7: Negative control. B. BQCV Sensitivity Analysis:M: 2000 bp DNA marker; 1: BQCV-10^5 copies/μL; 2: BQCV-10^4 copies/μL; 3: BQCV-10^3 copies/μL; 4: BQCV-10^2 copies/μL; 5: BQCV-10^1 copies/μL; 6: BQCV-10^0 copies/μL; 7: Negative control. C. DWV Sensitivity Analysis:M: 2000 bp DNA marker; 1: DWV-10^5 copies/μL; 2: DWV-10^4 copies/μL; 3: DWV-10^3 copies/μL; 4: DWV-10^2 copies/μL; 5: DWV-10^1 copies/μL; 6: DWV-10^0 copies/μL; 7: Negative control. D. IAPV Sensitivity Analysis:M: 2000 bp DNA marker; 1: IAPV-10^5 copies/μL; 2: IAPV-10^4 copies/μL; 3: IAPV-10^3 copies/μL; 4: IAPV-10^2 copies/μL; 5: IAPV-10^1 copies/μL; 6: IAPV-10^0 copies/μL; 7: Negative control.

Supplementary Figure 5 Specificity Validation Results

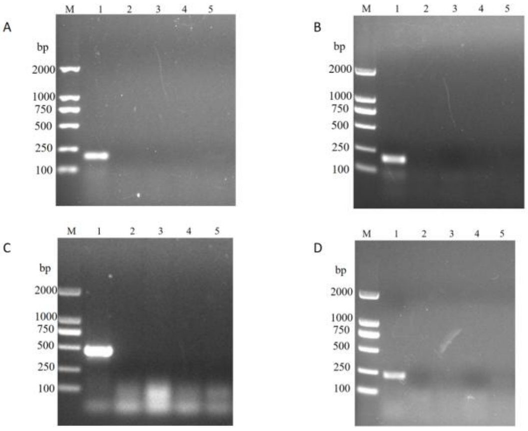

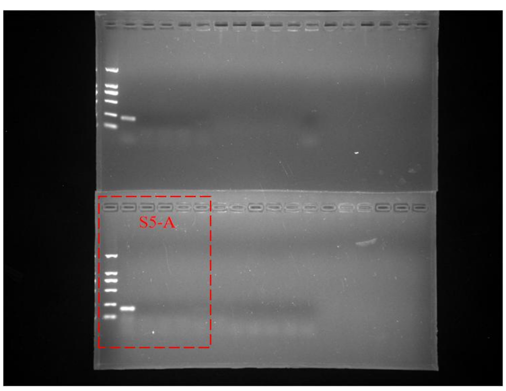

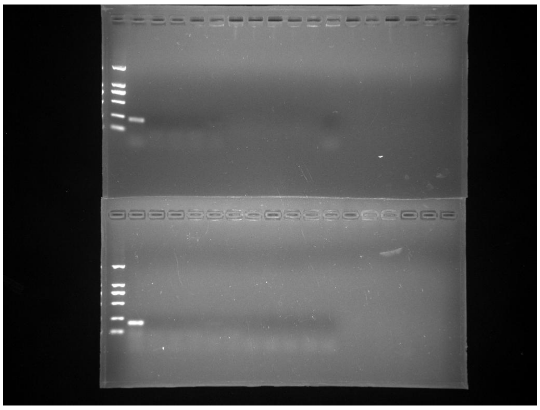

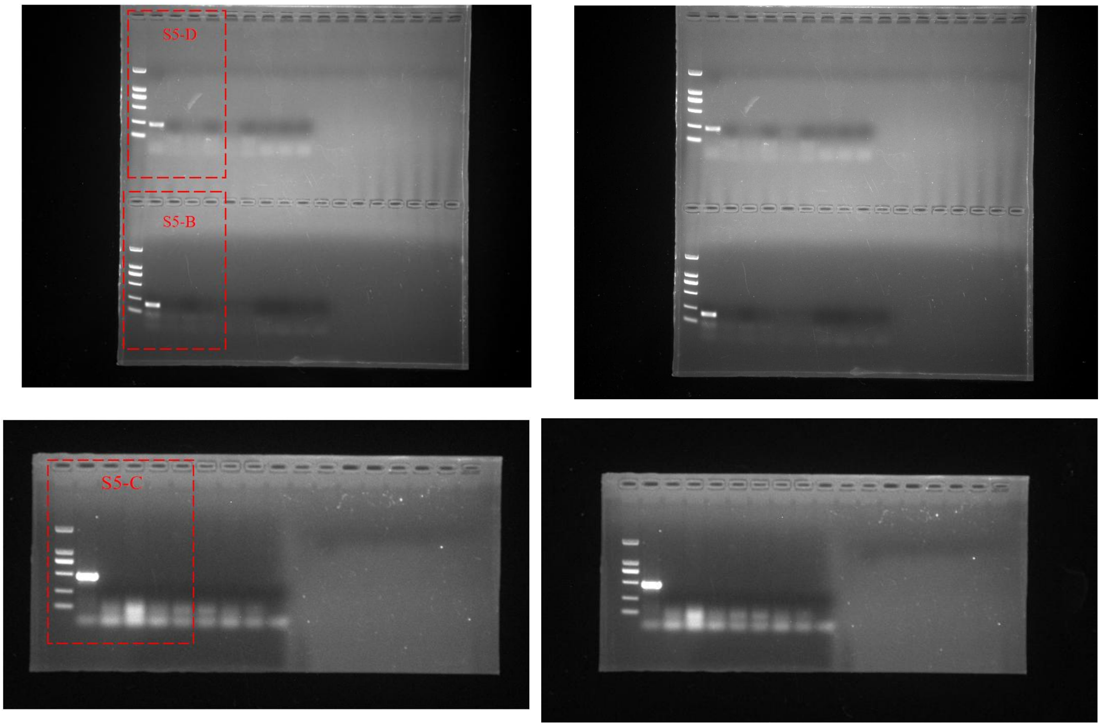

A. CBPV Specificity Validation:M: 2000 bp DNA marker; 1: CBPV; 2: BQCV; 3: DWV; 4: IAPV; 5: Negative control. B. BQCV Specificity Validation:M: 2000 bp DNA marker; 1: BQCV; 2: CBPV; 3: DWV; 4: IAPV; 5: Negative control. C. DWV Specificity Validation:M: 2000 bp DNA marker; 1: DWV; 2: CBPV; 3: BQCV; 4: IAPV; 5: Negative control. D. IAPV Specificity Validation:M: 2000 bp DNA marker; 1: IAPV; 2: CBPV; 3: BQCV; 4: DWV; 5: Negative control.

Figure 2:Sensitivity evaluation results of the PCR-LFD-MFCT system.

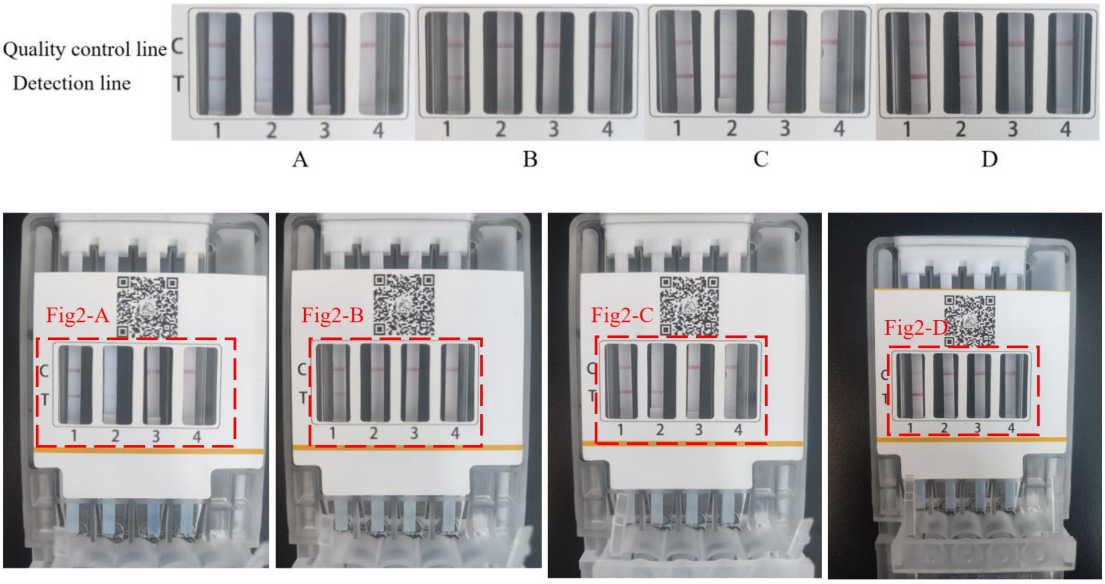

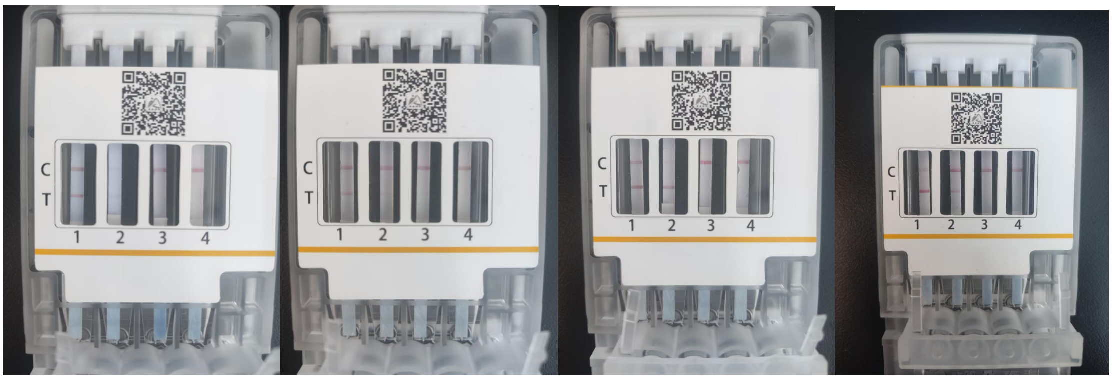

(Figure 2A) CBPV sensitivity: 1 : 1×103copies/μL; 2: 1×102copies/μL; 3: 1×101copies/μL; 4: Negativecontrol. (Figure 2B) BQCV sensitivity: 1: 1×103copies/μL; 2:1×102copies/μL; 3: 1×101copies/μL; 4: Negativecontrol. (Figure 2C) DWV sensitivity: 1: 1×103copies/μL ;2: 1×102copies/μL; 3: 1×101copies/μL; 4: Negativecontrol. (Figure 2D) IAPV sensitivity: 1: 1×103copies/μL ;2: 1×102copies/μL; 3: 1×101copies/μL; 4: Negativecontrol.

Figure 3:Specificity validation results of the PCR-LFD-MFCT system.

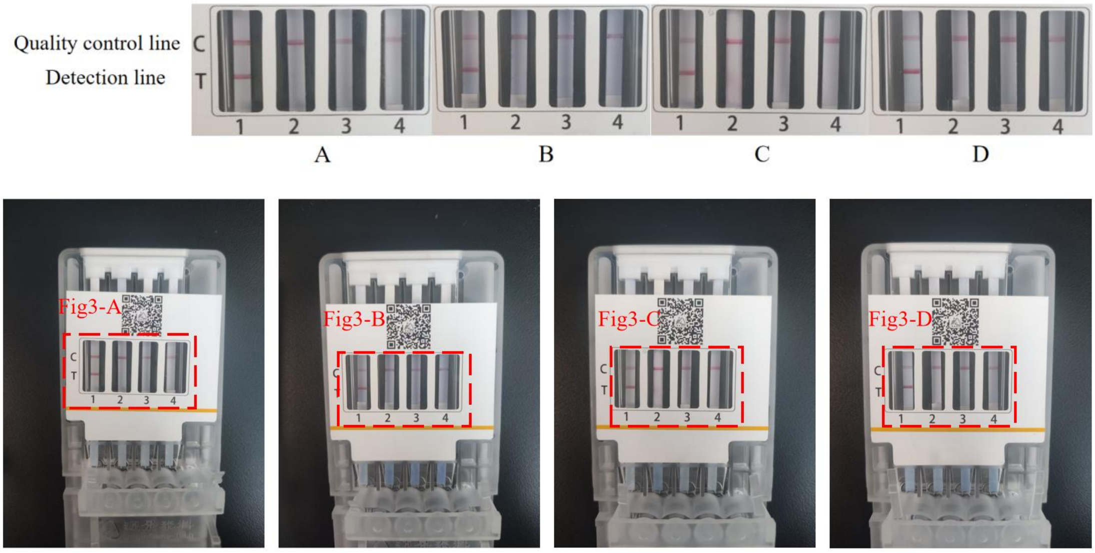

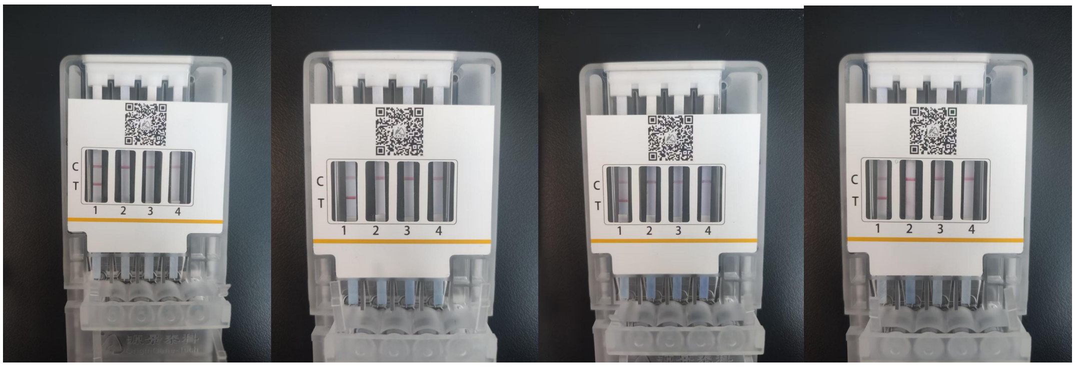

(Figure 3A)CBPV specificity: 1: CBPV; 2: BQCV; 3: DWV; 4: IAPV.(Figure 3B)BQCV specificity: 1: BQCV;2: CBPV;3: DWV;4: IAPV.(Figure 3C)DWV specificity: 1: DWV;2: CBPV;3: BQCV;4: IAPV.(Figure 3D)IAPV specificity: 1: IAPV;2: CBPV;3: BQCV;4: DWV.

Figure 4:Randomized testing of 12 simulated clinical samples with the PCR-LFD-MFCT system

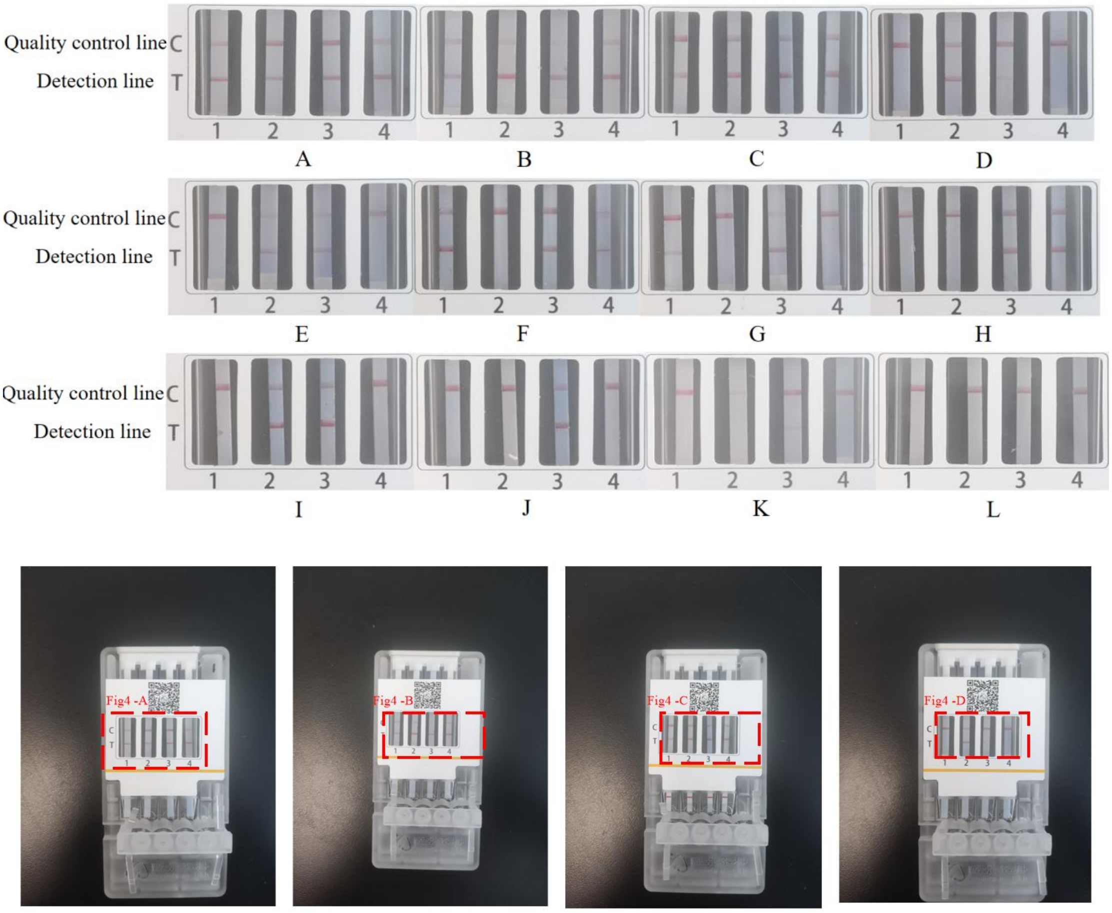

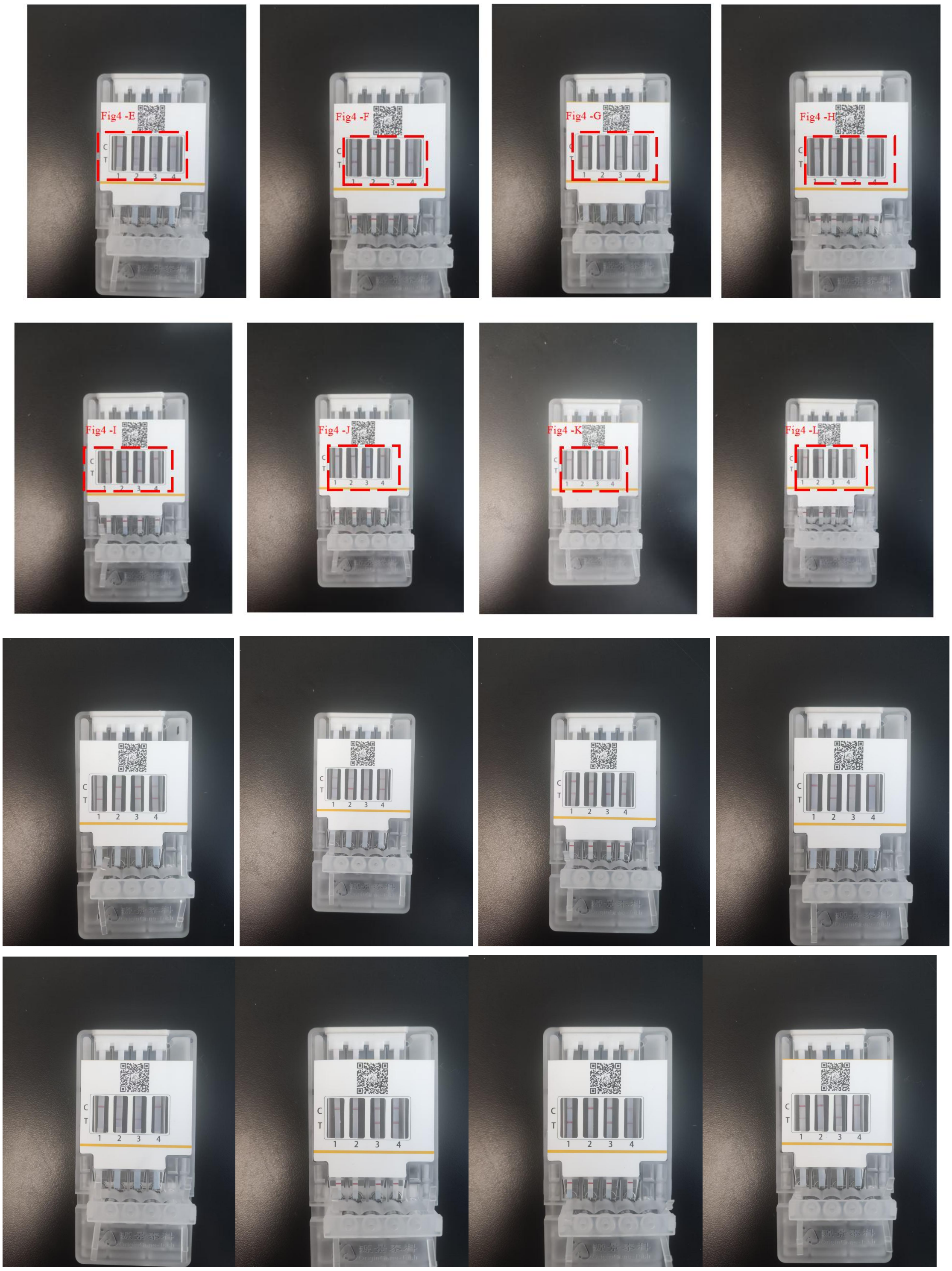
Fig4 -E
Fig4 -F
Fig4 -G
Fig4 -H
Fig4 -I
Fig4 -J
Fig4 -K
Fig4 -L

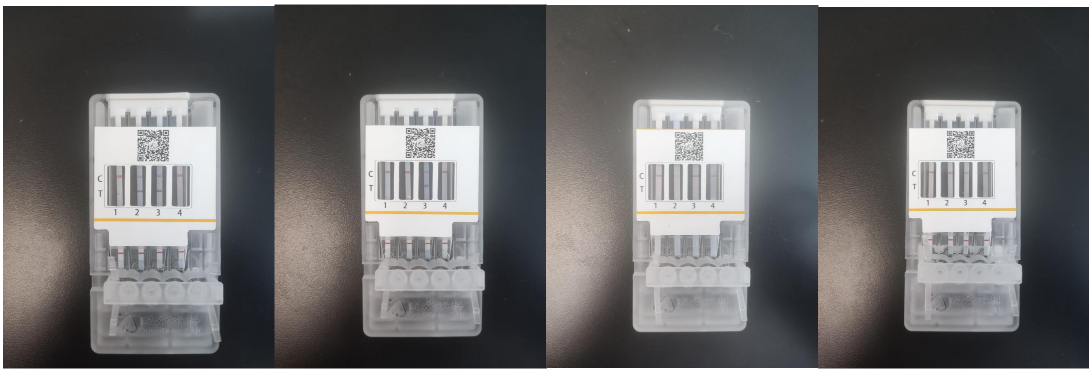

(Figure 4A-4L). 1: BQCV; 2: CBPV; 3: DWV; 4: IAPV.
