# Supplementary material for: Construction of a portable multiplex detection system for four bee viral paralysis diseases based on RT-PCR-microfluidic chip integrated technology
Source: BMC Vet Res. 2025 Aug 26;21:524. doi: 10.1186/s12917-025-04969-5 (PMC12379352; doi:10.1186/s12917-025-04969-5)
Supplement: Supplementary file 2 — Supplementary Material 2. [file 12917_2025_4969_MOESM2_ESM.doc]

Supplementary Table 1 Primer sequences for establishing the PCR detection method for CBPV

| Number | Name | Sequence（5'-3'） |
| --- | --- | --- |
| 1 | CBPV-F1  CBPV-R1 | AAACCTAGTAACATCGATCGAA  TTTGGGCTTCACAATAATCAGAT |
| 2 | CBPV-F2  CBPV-R2 | ACAATGCAAAACCTAGTAACATC  CCATCCCATTCTTTGGCAAAAT |
| 3 | CBPV-F3  CBPV-R3 | AAAACCTAGTAACATCGATCGA  TTTGGGCTTCACAATAATCAGA |
| 4 | CBPV-F4  CBPV-R4 | TTCCAGCAATGACAATGCAAAA  CATCCCATTCTTTGGCAAAATT |
| 5 | CBPV-F5  CBPV-R5 | CAATGCAAAACCTAGTAACATCG  ATCCCATTCTTTGGCAAAATTTCT |

Supplementary Table 2 Primer sequences for establishing the PCR detection method for BQCV

| Number | Name | Sequence（5'-3'） |
| --- | --- | --- |
| 1 | BQCV-F1  BQCV-R1 | TTACGGCAGTGAAGTCGCTC  AGCGATTGATGGAAGGAGGTG |
| 2 | BQCV-F2  BQCV-R2 | ATGCGCTTTATCGAGGAGGAG  TGGAACTCTGCGACTCCCTT |
| 3 | BQCV-F3  BQCV-R3 | GGAGATGTATGCGCTTTATCGAG  CACCAACCGCATAATAGCGATTG |
| 4 | BQCV-F4  BQCV-R4 | GCAAGCTCTTCCAATGATAG  AAGATTCAGCCGAGTCCTTA |

Supplementary Table 3 Primer sequences for establishing the PCR detection method for DWV

| Number | Name | Sequence（5'-3'） |
| --- | --- | --- |
| 1 | DWV-F1  DWV-R1 | TGTTTCGATTTTTGTGACTTTA  GCTAATTTTACCCAATCTTTAA |
| 2 | DWV-F2  DWV-R2 | GATTGTTTCGATTTTTGTGACTT  AATCTTTAAATTGTTTCGGTTTT |
| 3 | DWV-F3  DWV-R3 | GCGGTTACTTGAGAAATATCACT  TTGTTTCGGTTTTTGAGCAGCCA |
| 4 | DWV-F4  DWV-R4 | TACTTGAGAAATATCACTTGGC  ATTGTTTCGGTTTTTGAGCAGC |
| 5 | DWV-F5  DWV-R5 | TTTGCAAGATGCTGTATGTGG  GTCGTGCAGCTCGATAGGAT |

Supplementary Table 4 Primer sequences for establishing the PCR detection method for IAPV

| Number | Name | Sequence（5'-3'） |
| --- | --- | --- |
| 1 | IAPV-F1  IAPV-R1 | CCCACTTTGTATGGACACAATT  CATTTGCATATGCTCGGTCAAT |
| 2 | IAPV-F2  IAPV-R2 | CCACTTTGTATGGACACAATTCT  TCACATATAGTATTCCAGAAATC |
| 3 | IAPV-F3  IAPV-R3 | CCATGCCTGGTGATTCAC  CTGAATAATACTGTGCGTATC |
| 4 | IAPV-F4  IAPV-R4 | TTGTATGGACACAATTCTTGA  CACATATAGTATTCCAGAAAT |

Supplementary Table 5 Plasmid Construction Sequences

| BQCV、IAPV and DWV plasmids | CBPV plasmid |
| --- | --- |
| TAATACGACTCACTATAGGGGGAGATGTATGCGCTTTATCGAGGAGGAGTTCGAGTTAAAGTTGTTACTGAGAAGGGTGTAGATTTCGTCAGAGCTACCGTTAGTCCTCAACAGACTTACGGCAGTGAAGTCGCTCCTACTACTCATATCAGTACTCCTTTGGCAATAGAACAAATACCTATAAAGGGAGTCGCAGAGTTCCAAATACCGTACTATGCTCCATGTTTGTCATCTTCGTTTAGAGCGAATTCGGAAACATTTTACTATAGTTCAGGTCGGAATAATCTCGATATAGCCACTTCACCTCCTTCCATCAATCGCTATTATGCGGTAGGTGAGTTGTCATGGTTAACAGGATACGAGCGGTTCCTTGAGATCGATTTCGCTCGTTTCGACCAAACTCTGATGAAAGATCTCTTACGTATTGTCGAATTGAGGTTCTTACTCGACCCTTATACACCTAATCCTCACAACGACAATCACAACCAACGGGCCAACCAATTGTTCATCGCGTTTATGCTTTACACCCTTACCAACGTTGGTGTTAGTAGGTTTGGTACCCACTACAAGCGCGAAGGCACTCGTTGCTCAGGTGACCCTCACACCTCTATCGGCAACGGATTCATCAACGCATTCATCATTTGGCTCTGCCTTCGCAAACTGCCTACTAATAGCTGGCAGTCTGCTCATGAGGGTGACGACGGTATCGTTGGCTTGCGCGCTAACGTCGTCAATCAGGTCGAGTACAACCTCAGATTCCTCTCTTGTCTCGGCTTTCGTGCTAAGATTAGATAAATGGTTGCAAGATTAGAAGTTTGCAAGATGCTGTATGTGGTGTGCCTGGTTTAGATGGGTTTGATTCGATATCTTGGAATACTAGTGCTGGTTTTCCTTTGTCTTCATTAAAGCCACCTGGAACATCAGGTAAGCGATGGTTGTTTGACATTGAGCTACAAGATTCGGGATGTTATCTCCTGCGTGGAATGCGTCCCGAACTTGAGATTCAATTATCAACGACACAGTTAATGAGGAAAAAGGGAATAAAACCTCACACTATATTCACGGATTGTTTGAAAGATACTTGTTTGCCTGTTGAAAAATGTAGAATACCTGGCAAGACTAGAATATTTAGTATAAGTCCGGTACAGTTTACCATACCGTTTCGACAGTATTACTTAGACTTTATGGCATCCTATCGAGCTGCACGACCCATGCCTGGTGATTCACAACAAGAAAGCAATACTCCCAAGGTACACAATACGGAACTCGCTTCGTCCACTAGTGAAAACTCGATTGAGACTCAAGAAATCACAACCTTTCATGATGTGGAAACTCCAAATAGGATCGATACCCCCATGGCTCAGGATACTTCATCGGCTAGGAACATGGATGATACGCACAGTATTATTCAGAAAAAAAAAAAAAAAAA | AGCCTAAGCCCTTTCGTGACGGGCGCCCGATACTTGATGCCTGCCCTGACAACAGCGAACATAACAGTTCCAGCAATGACAATGCAAAACCTAGTAACATCGATCGAAACCAGAGTGATGGCTCCGACGCCTGCACTGCTGCCCTCGATAGCCTTTTTGGAACAACCCAGGGGACAGTATCTAGTAGCGATGCCCAACCTGCCTCAACACAGGCAACATCCAGCCAATGCGACCGGACTGGAGAAATTTTGCCAAAGAATGGGATGGGAAACGGAAATCATCCCGGAAGACCTGTTCACTCCGGAAATGGTTCATCAGACACCGAATCTGATTATTGTGAAGCCCAAAACCAAAAAAAAAAAAAAAAAAAAAAAAAA |

Supplementary Figure 1 Primer Screening Results


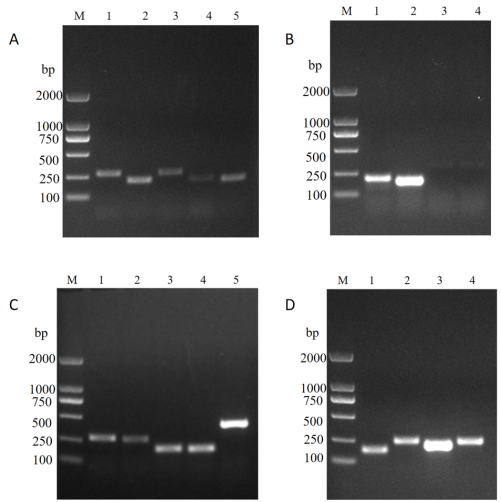


A. CBPV Primer Pair Screening:M: 2000 bp DNA marker; 1: CBPV-F1-R1; 2: CBPV-F2-R2; 3: CBPV-F3-R3; 4: CBPV-F4-R4; 5: CBPV-F5-R5. B. BQCV Primer Pair Screening:M: 2000 bp DNA marker; 1: BQCV-F1-R1; 2: BQCV-F2-R2; 3: BQCV-F3-R3; 4: BQCV-F4-R4. C. DWV Primer Pair Screening:M: 2000 bp DNA marker; 1: DWV-F1-R1; 2: DWV-F2-R2; 3: DWV-F3-R3; 4: DWV-F4-R4; 5: DWV-F5-R5. D. IAPV Primer Pair Screening:M: 2000 bp DNA marker; 1: IAPV-F1-R1; 2: IAPV-F2-R2; 3: IAPV-F3-R3; 4: IAPV-F4-R4.

Supplementary Figure 2 Primer Concentration Optimization Results


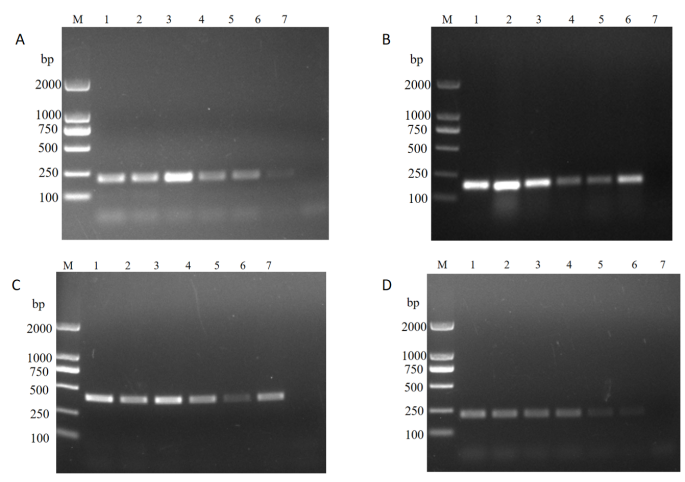


A. CBPV Primer Concentration Screening:M: 2000 bp DNA marker; 1: CBPV-14 μM; 2: CBPV-12 μM; 3: CBPV-10 μM; 4: CBPV-8 μM; 5: CBPV-6 μM; 6: CBPV-4 μM; 7: Negative control. B. BQCV Primer Concentration Screening:M: 2000 bp DNA marker; 1: BQCV-14 μM; 2: BQCV-12 μM; 3: BQCV-10 μM; 4: BQCV-8 μM; 5: BQCV-6 μM; 6: BQCV-4 μM; 7: Negative control. C. DWV Primer Concentration Screening:M: 2000 bp DNA marker; 1: DWV-14 μM; 2: DWV-12 μM; 3: DWV-10 μM; 4: DWV-8 μM; 5: DWV-6 μM; 6: DWV-4 μM; 7: Negative control. D. IAPV Primer Concentration Screening:M: 2000 bp DNA marker; 1: IAPV-14 μM; 2: IAPV-12 μM; 3: IAPV-10 μM; 4: IAPV-8 μM; 5: IAPV-6 μM; 6: IAPV-4 μM; 7: Negative control.

Supplementary Figure 3 Optimal Annealing Temperature Determination


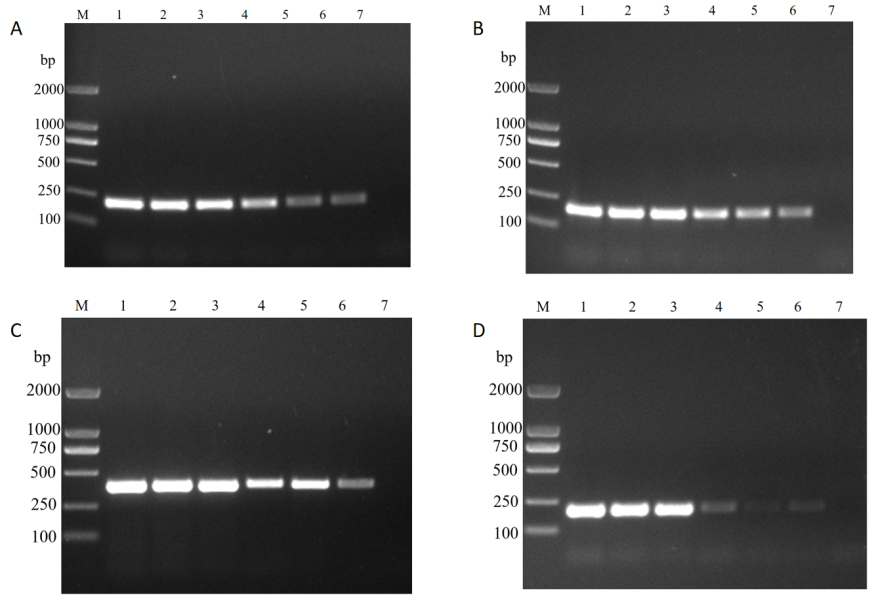


A. Optimal Annealing Temperature for CBPV:M: 2000 bp DNA marker; 1: CBPV-60℃; 2: CBPV-58℃; 3: CBPV-55℃; 4: CBPV-53℃; 5: CBPV-50℃; 6: CBPV-48℃; 7: Negative control. B. Optimal Annealing Temperature for BQCV:M: 2000 bp DNA marker; 1: BQCV-60℃; 2: BQCV-58℃; 3: BQCV-55℃; 4: BQCV-53℃; 5: BQCV-50℃; 6: BQCV-48℃; 7: Negative control. C. Optimal Annealing Temperature for DWV:M: 2000 bp DNA marker; 1: DWV-60℃; 2: DWV-58℃; 3: DWV-55℃; 4: DWV-53℃; 5: DWV-50℃; 6: DWV-48℃; 7: Negative control. D. Optimal Annealing Temperature for IAPV:M: 2000 bp DNA marker; 1: IAPV-60℃; 2: IAPV-58℃; 3: IAPV-55℃; 4: IAPV-53℃; 5: IAPV-50℃; 6: IAPV-48℃; 7: Negative control.

Supplementary Figure 4 Sensitivity Assessment Results


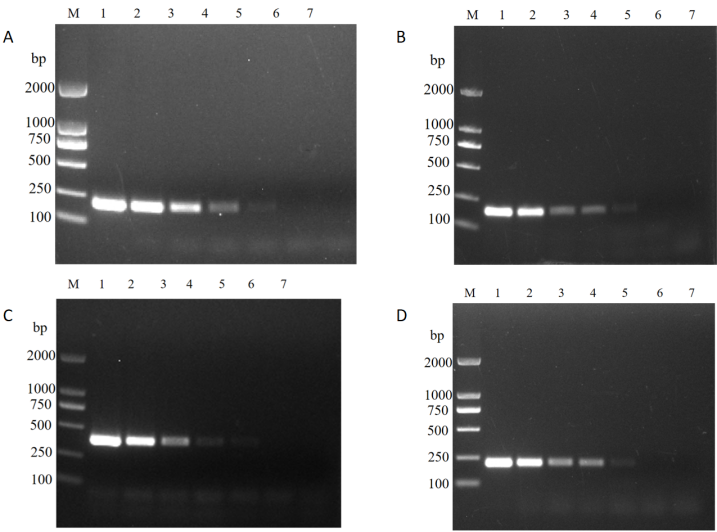


A. CBPV Sensitivity Analysis:M: 2000 bp DNA marker; 1: CBPV-10^5^ copies/μL; 2: CBPV-10^4^ copies/μL; 3: CBPV-10^3^ copies/μL; 4: CBPV-10^2^ copies/μL; 5: CBPV-10^1^ copies/μL; 6: CBPV-10^0^ copies/μL; 7: Negative control. B. BQCV Sensitivity Analysis:M: 2000 bp DNA marker; 1: BQCV-10^5^ copies/μL; 2: BQCV-10^4^ copies/μL; 3: BQCV-10^3^ copies/μL; 4: BQCV-10^2^ copies/μL; 5: BQCV-10^1^ copies/μL; 6: BQCV-10^0^ copies/μL; 7: Negative control. C. DWV Sensitivity Analysis:M: 2000 bp DNA marker; 1: DWV-10^5^ copies/μL; 2: DWV-10^4^ copies/μL; 3: DWV-10^3^ copies/μL; 4: DWV-10^2^ copies/μL; 5: DWV-10^1^ copies/μL; 6: DWV-10^0^ copies/μL; 7: Negative control. D. IAPV Sensitivity Analysis:M: 2000 bp DNA marker; 1: IAPV-10^5^ copies/μL; 2: IAPV-10^4^ copies/μL; 3: IAPV-10^3^ copies/μL; 4: IAPV-10^2^ copies/μL; 5: IAPV-10^1^ copies/μL; 6: IAPV-10^0^ copies/μL; 7: Negative control.

Supplementary Figure 5 Specificity Validation Results


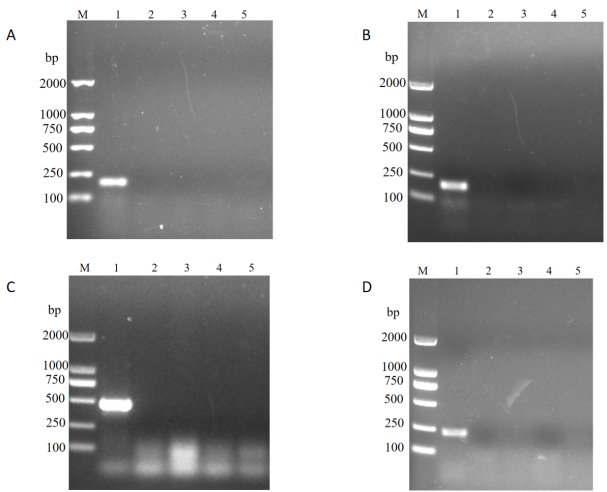


A. CBPV Specificity Validation:M: 2000 bp DNA marker; 1: CBPV; 2: BQCV; 3: DWV; 4: IAPV; 5: Negative control. B. BQCV Specificity Validation:M: 2000 bp DNA marker; 1: BQCV; 2: CBPV; 3: DWV; 4: IAPV; 5: Negative control. C. DWV Specificity Validation:M: 2000 bp DNA marker; 1: DWV; 2: CBPV; 3: BQCV; 4: IAPV; 5: Negative control. D. IAPV Specificity Validation:M: 2000 bp DNA marker; 1: IAPV; 2: CBPV; 3: BQCV; 4: DWV; 5: Negative control.
